# Reconfigurable all-dielectric metasurface based on tunable chemical systems in aqueous solution

**DOI:** 10.1038/s41598-017-03439-9

**Published:** 2017-06-09

**Authors:** Xiaoqing Yang, Di Zhang, Shiyue Wu, Yang Yin, Lanshuo Li, Kaiyuan Cao, Kama Huang

**Affiliations:** School of Electronics and Information Engineering, Sichuan University, Chengdu, 610065 China

## Abstract

Dynamic control transmission and polarization properties of electromagnetic (EM) wave propagation is investigated using chemical reconfigurable all-dielectric metasurface. The metasurface is composed of cross-shaped periodical teflon tubes and inner filled chemical systems (i.e., mixtures and chemical reaction) in aqueous solution. By tuning the complex permittivity of chemical systems, the reconfigurable metasurface can be easily achieved. The transmission properties of different incident polarized waves (i.e., linear and circular polarization) were simulated and experimentally measured for static ethanol solution as volume ratio changed. Both results indicated this metasurface can serve as either tunable FSS (Frequency Selective Surface) or tunable linear-to-circular/cross Polarization Converter at required frequency range. Based on the reconfigurable laws obtained from static solutions, we developed a dynamic dielectric system and researched a typical chemical reaction with time-varying permittivity filled in the tubes experimentally. It provides new ways for realizing automatic reconfiguration of metasurface by chemical reaction system with given variation laws of permittivity.

## Introduction

All-dielectric metamaterials were emerged as alternatives for constructing NRIM (Negative refractive index media) due to their advantages in low loss and symmetric design towards lossy metal-based structure. And their two dimensional analogues-metasurfaces were always used for adjusting the operating frequencies, transmissions and polarizations of EM wave.

The last decade, tunable metasurfaces for dynamic control EM wave propagation have gained some profound progress. They usually consist of a 2-D periodic array of resonator etched of conducting patches/strips printed on a dielectric substrate and modulatory elements. Several typical modulatory principles have been more recently considered to achieve tunability including tunable lumped elements^1, 2^, micromechanical devices^3, 4^, controllable liquid crystals^5, 6^, graphene^7, 8^, and microfluids/liquid metals^9, 10^. However, since the existence of metallic parts which were prone to breakdown, oxidization and corrosion, they can hardly be applied in complicated circumstances. Therefore, it is imperative to develop new types of tunable metasurfaces without using metallic structures.

The study of the application of tunable dielectric metamaterials well solved above-mentioned problems and were widely applied to frequency-selective surface^11^. Barton^12, 13^ developed genetic algorithms and fast Fourier transforms to generate random geometries and successfully tested the all-dielectric FSS. Meanwhile, Lepetit^14^ and Wang^15^ experimental realized left-handed metamaterial in all-dielectric composite cubes/rods. Li^16^ proposed all-dielectric metamaterial FSS at microwave frequencies based on high-permittivity ceramic resonators. By adjusting the geometrical parameters of the ceramic resonator, it can inspire the desired resonant modes for designing tunable or reconfigurable metamaterials. Li^17^ further realized reconfigurable EM responses between two adjacent stopbands by mechanically tuning the orientation of the ceramic resonators. With the development of all-dielectric metamaterials, tunable or reconfigurable methods were further proposed and realized toward high-frequency applications in terahertz^18, 19^, infrared^20, 21^ and even optical regions^22^. However, there always exist some defects of these reconfigurable methods, such as high cost, hard processing and difficult to operate in practice, etc.

Until recently, water, a neglected material, has been employed for negative-index optical metamaterials^23^, acoustic metamaterials^24^ and EM metamaterials^25^. Andryieuski^26^ proposed to use the properties of water for tunable transmission of EM waves through a system of partially filled water meta-atoms. Tunability was provided by the gravity force, which readily reshaped the water volume in each elementary reservoir with rotation of the whole system of the meta-atoms. In such configuration, the water-based metamaterials can be definitely considered as the simplest and potentially the cheapest realization of a tunable transmission of EM waves. Then Mikhail Odit^27^ provided the experimental demonstration of this functional device. However, its tunable range is limited to the rotated angle of 90° and it is not applicable for high-efficiency cross-polarization wave conversions, as well as for linear-to-circular polarization wave conversions.

In this paper, we proposed a novel reconfigurable method of all-dielectric metasurface by the “chemical ways”, which made use of the most common nature-friendly material, namely, water and its solution. Not only its superiority of abundance and low price but also the functionality is typically high-permittivity, which serves to compensate the lack of free carriers by employing Mie resonances^28, 29^. Therefore, the fabricated metasurface is cheap, easy processing and in good operability. The operating frequencies, transmissions and polarizations can be modulated by injection of different volume ratio of mixture solution, which is equivalent to changing the complex permittivity. Based on calculated mixture permittivities and experimental measured results, a simplified calculated model was derived for predicting the maximum magnitudes and their corresponding frequency shifts that related changes of complex permittivity.

According to three filled states of two oriented tubes, the metasurface shows tunable pass-band characteristics for incidence of y- or x-polarization wave, as well as tunable linear-to circular polarization or cross-polarization conversion characteristics at required frequency. In addition, we developed the chemical reaction with time-varying complex permittivity as reconfigurable mechanism and experimentally demonstrated the transmission curves can spontaneously change as the time progresses without extra control system. This principle provides new ways for realizing automatic reconfiguration of metasurface by chemical reaction systems with given variation laws of permittivity. Hence, the proposed metasurface is easily reconfigurable and multifunctional, which provides new ways to manipulate EM wave propagation.

## Methods

### The design theory of all-dielectric metasurface

High-permittivity dielectric metamaterials working in the microwave region, can be designed by effective medium theory and dielectric resonant theory. Due to the open circuit state at the dielectric/air interface, when EM waves enter the resonators, they are reflected on the inside walls of the dielectric particle and the power can be forced inside the dielectric by the high-permittivity. This forms standing waves and results in different resonant modes inside the dielectric resonator^16^. In fact, the transmission characteristics of metasurface are mainly determined by the effective impedance of the metasurface, which is, matching in the pass-band while mismatching in the stop-band. Thus, the transmission characteristics of all-dielectric metasurface can be adjusted via the resonant modes of the dielectric resonators^17^. To design FSSs with pass-band or stop-band in the considered frequencies, tuning complex permittivity of dielectric resonators is equivalent to tune the effective EM impedance. Thus, in the framework, all-dielectric FSSs can be well designed. Meanwhile, rotation of this metasurface would have different effect on the circular polarized wave transmissions and conversions. Such behavior towards circular polarization can be used for designing Polarization Converters. This can be explained by the bianisotropy of the metasurface. However, detailed characterization of the bianisotropic properties of the metasurface is beyond the scope of the Letter.

### Simulation and measurement

**Figure 1:**
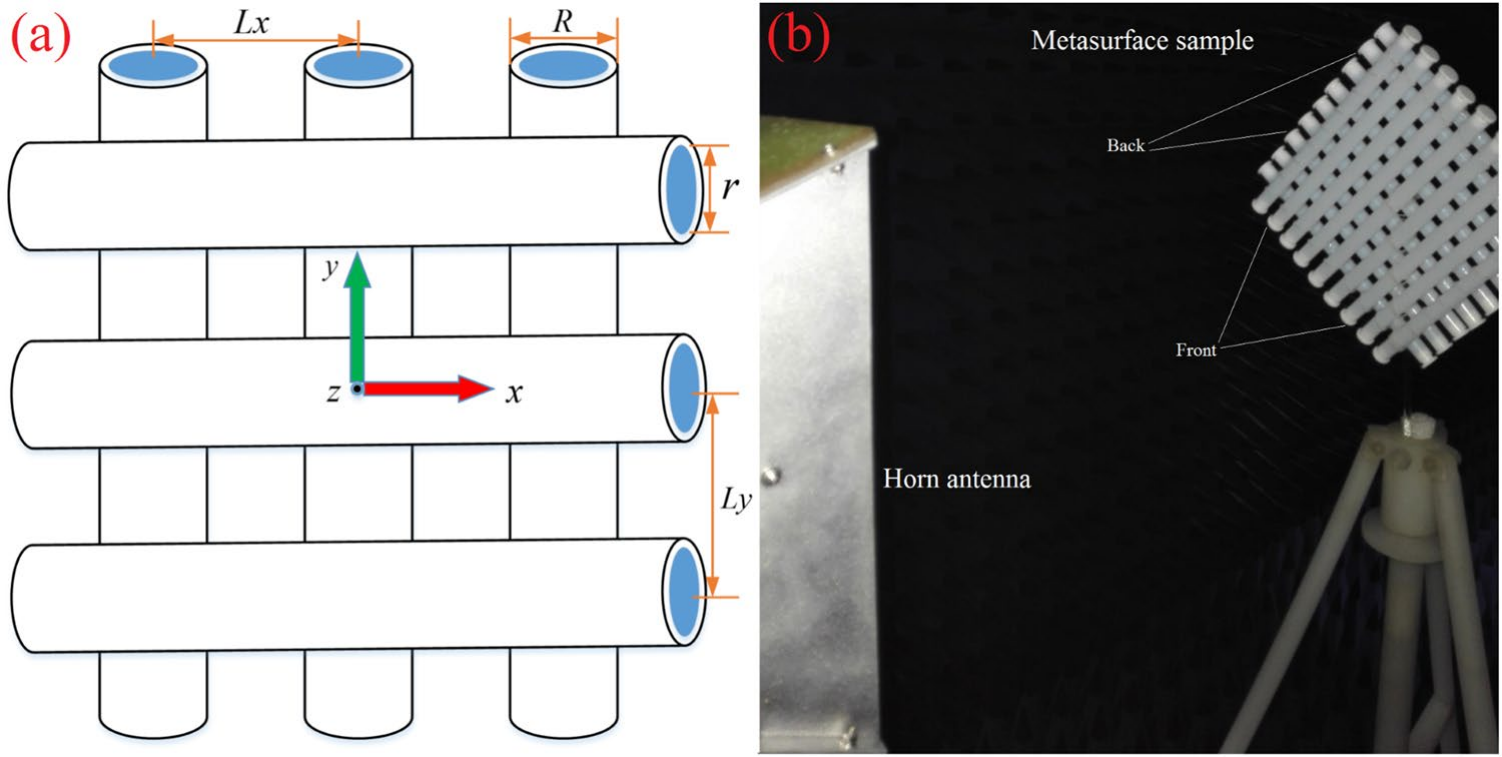
(**a**) Schematic of the unit cell of teflon tubes. (**b**) Photograph of the experimental setup.

The proposed metasurface is composed of teflon tubes periodically arranged in two orthogonal directions, paralleled to the x-axis and y-axis separately, as seen in Fig. 1(a). The outer and inner diameter of teflon tubes are R = 6 *mm* and r = 5 *mm*, while the intervals of adjacent tube are *Lx* = 20 *mm* and *Ly* = 20 *mm* respectively. The inner materials of tubes are simply filled with different aqueous solution for reconfiguration. Full-wave simulation of the metasurface transmission coefficient upon the plane-wave excitation was conducted using the frequency domain of CST Microwave Studio solver. Two Bloch-Floquet ports along the z direction in the far-field were used. And the unit cell periodic boundary conditions were imposed for single element, so that the structure and the incident wave are periodically repeated along x- and y-axis. To demonstrate the reconfiguration, the corresponding theoretical value of complex permittivity for different mixture solution was substituted into simulation.

The prototype of the tunable aqueous solution metasurface was fabricated manually, without complicated and high-precision processing, and experimentally investigated in an anechoic chamber, as shown in Fig. 1(b). There are 9 experimental tubes placed on the opposite sides. The front and back tubes are respectively closed to transmitting and receiving antennas. And the sample occupies an area of 200 *mm* × 200 *mm*. Each termination was blanked off by rubber plug and easily opened up for injection of aqueous solution. All tubes were filled up by injection of replaceable mixture solutions. The metasurface was supported by antenna pedestal with a stationary glass rod. A pair of identical rectangular linear polarized broadband horn antennas (180 × 140 *mm* ^2^) connected to the coaxial ports of an Agilent E8363C Vector Network Analyzer was used to perform emission and detection of plane wave. Two LCP-antennas and one RCP-antenna made of helical antennas^30^ (maximum diameter is 75 *mm*) were fabricated in order to perform and separate circular polarization characterization. Antennas were matched in the frequency range of 2–3 GHz. The measured metasurface was located at the distance of 1 *m* to both the transmitting and receiving antennas, which meet the far-field condition.

### Complex permittivity of chemical systems in aqueous solution

The ***ε*** can be expressed by the formulation:

1 $$\varepsilon =\varepsilon ^{\prime} -j\varepsilon ^{\prime\prime} $$

where *ε*′ and *ε*″ are the real and imaginary part of the complex permittivity, which respectively affects the resonant properties and reveals the loss of microwave energy.

Based on our previous researches in characterization of dielectric properties in materials^31^, mixture solution^32^ and reaction system^33, 34^, we divided the tunable chemical systems into two parts, static mixture aqueous solutions (time-invariant permittivity) and dynamic chemical reactions (time-varying permittivity).

For static mixture aqueous solutions, we theoretically calculated mixture effective permittivity (***ε***_***eff***_) of anhydrous ethanol (***ε***_***i***_ = *7.4581* + *j6.6545*) and ultra-pure water (***ε***_***e***_ = *73.1202* + *j1.4143*) in different mixture proportion by Bruggeman formula^35^,

2 $$\frac{{\varepsilon }_{eff}-{\varepsilon }_{e}}{{\varepsilon }_{eff}+2{\varepsilon }_{e}+v({\varepsilon }_{eff}-{\varepsilon }_{e})}=\alpha \frac{{\varepsilon }_{i}-{\varepsilon }_{e}}{{\varepsilon }_{i}+2{\varepsilon }_{e}+v({\varepsilon }_{eff}-{\varepsilon }_{e})}$$

Where ***α*** denotes volume ratio of ethanol/ultra-pure water, ***v*** equals to 2 in the case of two mixture solutions. Fortunately, though the solutions are typically frequency-varying media, their permittivities are slightly linear change within the range of 2–3 GHz. For simplification, the frequency-varying permittivity in total range can be equivalent to the constant values under 2.45 GHz which is always used for permittivity measurement. As seen in Table 1, we have calculated the complex permittivity and their difference of five groups for simulations and experiments, where ${\rm{\Delta }}\varepsilon ^{\prime} ={\varepsilon }_{mix}^{^{\prime} }-{\varepsilon }_{e}^{^{\prime} }$, ${\rm{\Delta }}\varepsilon ^{\prime\prime} ={\varepsilon }_{mix}^{^{\prime\prime} }-{\varepsilon }_{e}^{^{\prime\prime} }$, with subscript (*mix*) for the mixture solution and (*e*) for the ultra-pure water. Notably, volume ratio is defined as ethanol over ultra-pure water through full paper.

**Table 1:** The calculated value and their difference in different volume ratio of ethanol/ultra-pure water (2.45 GHz, 25 °C).

Volume ratio (ethanol/ultra-pure water)	*ε*′	*ε*″	*ε*′	*ε*″
0:1	73.1202	1.4143	0	0
1:25	69.5485	1.8569	−3.5717	0.4426
1:10	64.7185	2.4786	−8.4017	1.0643
1:6	59.9898	3.1135	−13.1304	1.6992
1:4	54.8673	3.8297	−18.2529	2.4154

As for dynamic chemical reactions in aqueous solution, most researches have manifested the complex permittivity varies with reaction time due to the concentration change of reactants and products or temperature variation of reaction system. The real part and imaginary part of complex permittivity often appear different variable laws in terms of various reaction types. This principle is expected to apply for realizing automatic reconfiguration of metasurface.

## Results

Due to the periodic structure that is constructed by front and back array tubes, different filled states of two oriented tubes would emerge heterogeneous EM responses. Considering the symmetries, we manually divided the filled states into three types for static mixture solutions:

State 1: Same aqueous solutions were filled both in front and back tubes.

State 2: Variable aqueous solutions just filled in back tubes while front tubes keep filled ultra-pure water.

State 3: Variable aqueous solutions just filled in front tubes while back tubes keep vacuum.

Besides, dynamic chemical reaction system with time-varying permittivity filled in the State 3 was experimentally researched.

**Figure 2:**
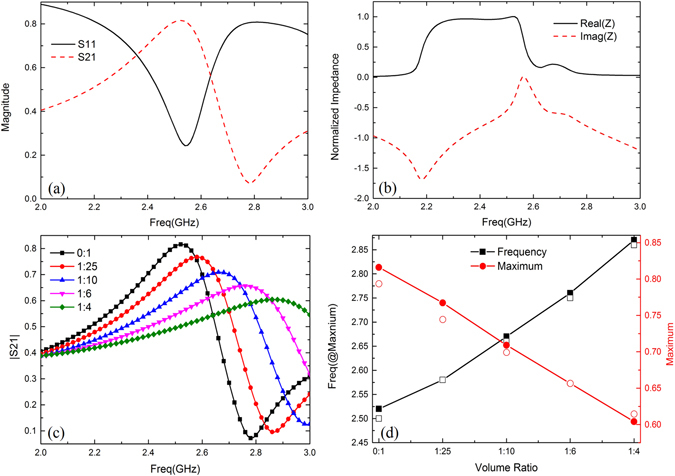
(**a**) Simulated transmission and reflection for filled ultra-pure water in dB. (**b**) The normalized impedance for filled ultra-pure water. (**c**) Simulated transmissions for state 1. (**d**) Simulated (solid) and measured (open) maximum magnitudes and their corresponding resonant frequencies vary with volume ratio for state 1.

**State 1**. Figure 2 gives an example to explain the resonance characteristics firstly, and then shows the tunability of transmissions varied with volume ratio. Figure 2(a) presents the simulated transmission/reflection magnitudes of filled ultra-pure water for normally incident of y-polarized plane waves. There is transmission dip at about 2.78 GHz, which indicates the microwave cannot propagate. The normalized impedance^36^ shown in Fig. 2(b) can explain why the stop-band forms. From 2.2 GHz to 2.6 GHz, the normalized impedance is 1, which indicates the system is in good match, so the metasurface is transparent to EM waves. After that, this value drops to the minimum due to the strong electric resonance, the impedance matching becomes worse, resulting in reduced transmission. The origin of the resonance can be further explained using the dielectric resonator theory. When EM waves enter the metasurface, they are reflected upon the dielectric-air interfaces, which forms standing waves. The field distribution inside the high-permittivity dielectric can be equivalent to an electric dipole and result in electric resonance. Figure 2(c) shows all transmissions reach the maximum firstly and then drop to the minimum. The peak magnitudes are downward while the corresponding resonant frequencies are upward with the increase of volume ratio. Moreover, the calculated insertion loss is extended from 1.1 dB to 6 dB in the pass-band. The insertion loss can be improved by employing low loss solvent, such as methanol, acetic acid and so on. Figure 2(d) shows maximum magnitudes and their corresponding frequencies varied linearly with volume ratio.

Thus, a simplified predictive model that relates changes in the complex permittivity to frequency and maximum shifts can be derived with the respective following matrix form^37^ as

3 $$[\begin{array}{c}{\rm{\Delta }}{f}_{0}\\ {\rm{\Delta }}|S21|\end{array}]=[\begin{array}{cc}{m}_{11} & {m}_{12}\\ {m}_{21} & {m}_{22}\end{array}]\,[\begin{array}{c}{\rm{\Delta }}\varepsilon ^{\prime} \\ {\rm{\Delta }}\varepsilon ^{\prime\prime} \end{array}]$$

where $${\rm{\Delta }}{f}_{0}={f}_{mix}-{f}_{e}$$, $${\rm{\Delta }}|S21|={|S21|}_{mix}-{|S21|}_{e}$$.

The coefficients of the model in (2) are over determined by a set of linear measured datasets in Table 2. The least-squares method explained in ref. 36 can be used to approximate the interdependency as

4 $$[\begin{array}{c}{\rm{\Delta }}{f}_{0}\\ {\rm{\Delta }}|S21|\end{array}]=[\begin{array}{ll}-{\rm{0.0149}} & {\rm{0.0353}}\\ \,{\rm{0.0474}} & {\rm{0.2869}}\end{array}]\,[\begin{array}{c}{\rm{\Delta }}\varepsilon ^{\prime} \\ {\rm{\Delta }}\varepsilon ^{\prime\prime} \end{array}]$$

**Table 2:** Measured, calculated variations and their relative errors.

*Measurement*		*Calculation (Prediction Model)*		|*Relative Errors*|	
Δ*f*_*0*_	Δ|*S21*|	Δ*f*_*0*_	Δ|*S21*|	Δ*f*_*0*_	Δ|*S21*|
0.08	−0.0475	0.0688	−0.0422	15%	11.15%
0.16	−0.09096	0.1627	−0.0925	1.68%	1.69%
0.25	−0.13204	0.2555	−0.1343	2.2%	1.71%
0.36	−0.17269	0.3570	−0.1714	0.83%	0.074%

The relative errors refer to the differences between calculation and measurement divided by calculated values.

By substituting the complex permittivity from Table 1 into predictive model, the calculated variations are shown in Table 2 as well as the relative error. With the variations of complex permittivity increasing, the absolute values of relative error between measurements and calculations get smaller. It indicates that the predictive model is more accurate for significant change of complex permittivity. The similar predictive model can be founded for state 2 and state 3 by the above-mentioned method. There is no need to repeat in the following sections.

**Figure 3:**
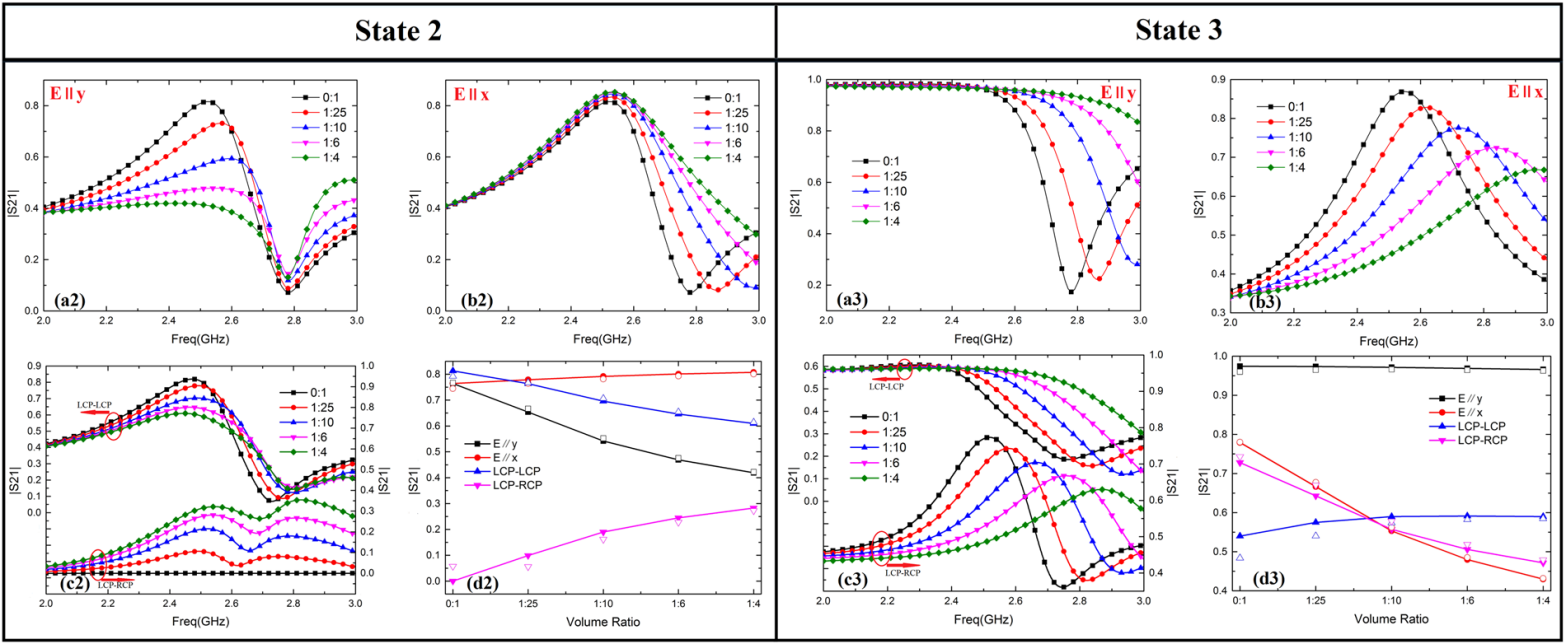
(**a2**,**a3**) Simulated transmissions of y-polarization for state 2 and state 3. (**b2**,**b3**) Simulated transmissions of x-polarization for state 2 and state 3. (**c2**,**d2**) Simulated conversions of LCP-LCP and LCP-RCP for state 2 and state 3. (**d2**,**d3**) Simulated (solid) and measured (open) magnitudes vary with volume ratio at 2.45 GHz for state 2 and state 3.

**State 2**. The front tubes are set to parallel to x-axis. Figure 3 shows the reconfigurable transmission properties of linear polarization (E∥y denotes y-polarization and E∥x denotes x-polarization) and circular polarization (LCP denotes Left Circular Polarization and RCP denotes Right Circular Polarization). Figure 3(a2,b2) show the variable transmissions of y-polarization and x-polarization respectively. Both of them reach the maximum firstly and then drop to the minimum. The differences between them are the former keep all the minimums at 2.78 GHz while the latter maintain all the maximums at 2.54 GHz, no matter what the volume ratios are. By increasing the volume ratio of ethanol, the transmission characteristics can be easily reconfigurable, realizing the increase of insertion loss and the bandwidth for incidence of different polarization waves. Figure 3(c2) shows the cross-polarization (LCP-RCP) transmissions are under 0.4 and co-polarization (LCP-LCP) transmissions achieve up to 0.78. It suggests that the metasurfaces have strong ability for circular polarization-conserving transmittances at required frequency. Simulated and measured magnitudes are compared in Fig. 3(d2) at 2.45 GHz and they are in good agreement. Transmissions of different incident polarized wave monotonously change with volume ratio respectively and the similar predictive model can be founded by the same way illustrated in State 1.

**State 3**. Likewise, the front tubes are set to parallel to x-axis. Figure 3(a3) shows transmissions of y-polarization stay at 0.98 below 2.5 GHz, then drop quickly and split due to transmission characteristics of different permittivity. By tuning the mixture ratio, the metasurfaces can serve as tunable low-pass FSS (Frequency Selective Surface). Figure 3(b3) shows transmissions achieve maximum firstly, and then drop to the minimum. Differently, they work as tunable band-pass FSS. Figure 3(c3) shows the co-polarization transmissions are under 0.6 while cross-polarization transmissions achieve up to 0.78. It indicates that the metasurfaces have strong ability for cross-polarization conversions at required frequency. Simulated and measured magnitudes are compared in Fig. 3(d3) at 2.45 GHz and they are in good agreement. Similarly, it can be good linear fitting for predictive model at specific frequency.

The PCR (Polarization Conversions Ratio) is defined to represent the ratio of cross-polarization wave in total transmitted wave,

5 $$PCR=\frac{{T}_{LR}^{2}}{({T}_{LL}^{2}+{T}_{LR}^{2})}$$

*T*_*LR*_ represents transmission of LCP-RCP, *T*_*LL*_ represents transmission of LCP-LCP. The measured PCRs are shown in Table 3 for state 2 and state 3. The cross-polarization conversion ratios nearly monotonously change with volume ratio. It suggests that the metasurfaces can be reconfigurable for high-efficiency tunable Polarization Converter for cross-polarization conversions at required frequency by tuning the volume ratio or filled state at specific frequency.

**Table 3:** The PCRs of Cross-Polarization at 2.45 GHz and 2.78 GHz for state 2 and state 3.

State/Polarization	Volume Ratio PCR	0:1	1:25	1:10	1:6	1:4
**State 2**	2.45 GHz	0.00523	0.00536	0.04999	0.10818	0.16433
	2.78 GHz	0.05487	0.1223	0.60337	0.68925	0.80095
**State 3**	2.45 GHz	0.70226	0.60607	0.50417	0.44231	0.40164
	2.78 GHz	0.82435	0.89172	0.84145	0.75659	0.62507

**Figure 4:**
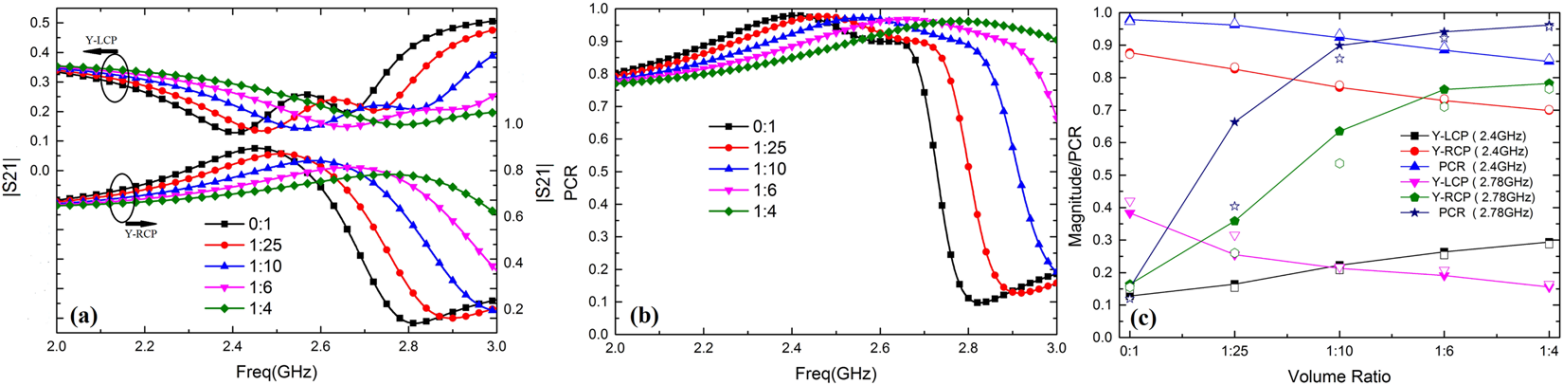
(**a**) Simulated transmission of Y-CP (**b**) Calculated PCRs vary with volume ratio (**c**) Simulated (solid) and measured (open) magnitudes vary with volume ratio at 2.4 GHz and 2.78 GHz respectively.

For charactering its ability of LP-CP (linear-to-circular polarization), the sample was anticlockwise rotated 45 degree around z-axis as shown in Fig. 1(b). Figure 4(a) presents the reconfigurable transmissions of LP (take y-polarization as example) to CP. The Y-LCP transmissions firstly decrease to the minimum and then increase while the Y-RCP transmissions behave in the opposite way. The metasurfaces serve as Polarization Converter which high-efficiently convert y-polarization to RCP. With the increase of volume ratio, the transmission maximums decline while the pass-bands are broadened. Figure 4(b) gives the PCRs (here we denote Y-LCP as TLR for calculations) in the whole frequency range. One can see that the converter efficiency is extended from 0.12 to 0.98. The workable bandwidth is broadened with increase of volume ratio, even out of the measured frequency range. As seen in Fig. 4(c), the transmissions of different polarized waves and their PCRs change significantly with volume ratio. At 2.4 GHz, the magnitudes of Y-LCP keep small but increase with the volume ratio, while the magnitudes of Y-RCP show the opposite trends. At 2.78 GHz, the Y-LCPs drop slightly but Y-RCPs rise significantly with the volume ratio increasing. Then we consider the PCRs, which both drastically rise at two different frequency. It demonstrates the metasurfaces can be easily reconfigurable for high-efficiency tunable linear to circular polarization conversions by tuning the volume ratio.

### Chemical reaction system

Since the mixture solutions are time-invariant, it is hard to realize automatic time-varying reconfiguration of metasurface by mechanically modulating. Therefore, it is necessary to develop a time-varying tunable system filled in the tubes for realizing continuously and automatically reconfigurable metasurface. The chemical reaction in aqueous solution is a typical time-varying permittivity system, while the variation laws of permittivity can be artificially set by different initial concentrations or reaction conditions. Once we obtained the variation laws of various reaction types, the ‘chemical metasurface’ can be applied in specific controlled situation, and meet different requirements of automatic modulation. This principle is expected to provide new ways for realizing automatic reconfigurable metasurface by using chemical reaction system with specific variation laws of permittivity.

In order to prove the feasibility, we selected two typical reactions whose variation laws of permittivity were obtained^33^. The reaction equations are saponification reaction

6 $${{\rm{CH}}}_{5}{{\rm{COOC}}}_{2}{{\rm{H}}}_{5}+{\rm{NaOH}}\to {{\rm{CH}}}_{5}{\rm{COONa}}+{{\rm{C}}}_{2}{{\rm{H}}}_{5}{\rm{OH}}$$

and acetone iodination reaction^34^

7 $${{\rm{KIO}}}_{3}+5{\rm{KI}}+6{\rm{HCL}}+3{{\rm{CH}}}_{3}{{\rm{COCH}}}_{3}\to 3{{\rm{CH}}}_{3}{{\rm{COCH}}}_{2}{\rm{I}}+6{\rm{KCL}}+3{\rm{HI}}+3{{\rm{H}}}_{2}{\rm{O}}$$

**Figure 5:**
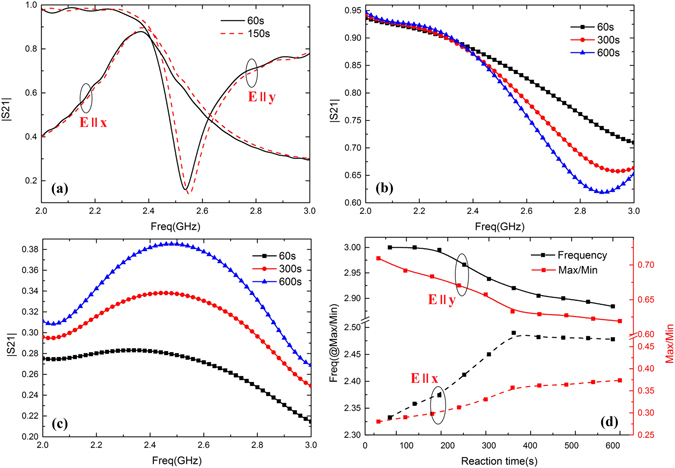
(**a**) Measured transmission of y-polarization and x-polarization for saponification reaction. (**b**) Variable transmissions of y-polarization for acetone iodination reaction. (**c**) Variable transmissions of x-polarization for acetone iodination reaction (**d**) Measured data for dips or peaks magnitudes and their corresponding frequencies over time.

The initial concentrations are same as references and the reaction was performed under 25 °C. The measurement results of the transmissions at various frequencies for y- and x-polarization are shown in Fig. 5. The reactants were fast injected and measurements were conducted after 60 s. Data are collected every 10 s, but only a few curves representing sample data are plotted. The curves illustrate how the transmissions change as the time progresses, which corresponds to the change of the effective permittivity of the solution during the reaction. For saponification reaction (6), the shifts of transmission curves are relative small due to the imperceptible changes of permittivity, as shown in Fig. 5(a). Such a slow change of less than 5% cannot be any useful in the practical applications. Therefore, we further introduced the acetone iodination reaction (7) with significant changes of permittivity over time and measured the transmissions. Figure 5(b,c) show variable transmissions of y-polarization and x-polarization respectively with reaction time. The transmission dips are downward in Fig. 5(b) while the peaks are upward in Fig. 5(c) as the time progresses. Figure 5(d) shows 10 sets of measured data for dip or peak magnitudes and their corresponding frequencies over time. In the case of different incidences, the curves present different variation trends for about 400 s. Finally, they remain nearly constant due to stop of reaction. Similarly, other chemical reaction can be designed deliberately and meet different reconfiguration requirements by tuning the concentration of reactants or temperature of reaction system. This principle provided new reconfigurable ways for wireless communication system by using chemical reaction system with given variation laws of permittivity.

## Discussion

We proposed and fabricated reconfigurable all-dielectric metasurface consisting of cross-shaped periodic teflon tubes based on mixture solution or chemical reaction. The numerical simulations and experimental measurements proved that either linear polarization or circular polarization wave can be easily modulated by tuning the complex permittivity of chemical systems. According to three filled states of two oriented tubes, we demonstrated the proposed metasurfaces can either sever as tunable FSS or Polarization Converter. The metasurface array was linear polarization angle dependent, which shows tunable pass-band characteristic for incidence of y- or x-polarization. Moreover, the proposed metasurface can dynamically achieve high-efficiency linear-to-circular/cross polarization conversions at required frequency range. Furthermore, we developed chemical reaction with time-varying complex permittivity as tunable mechanism and experimental demonstrated the ‘chemical metasurface’ can automatically modulate of EM wave without extra control system. It provides new ways for realizing automatic reconfigurable metasurface by using chemical reaction system with given variation laws of permittivity. The potential of automatic modulation can be further studied by other reaction types, such as Belousov-Zhabotinsky reaction, which serves as a classical example of non-equilibrium thermodynamics, resulting in the establishment of a nonlinear chemical oscillator and realizing of periodically automatic reconfiguration. The main advantages of proposed metasurface are cheap, convenient and easily reconfigurable, as well as multi-functional for polarized wave modulation and conversion. It has the potential to realize automatic reconfiguration and will has promising future in wireless communication systems such as RCS reduction techniques and reconfigurable antenna systems.

Similar reconfigurable concepts can be applied to metamaterial absorber, especially high-cost electrolyte solution that is naturally high-absorption for high-frequency application. It has potential to realize reconfigurable absorber of broadband in various products including transparent and conformal materials. Using the plasticity of liquid materials, the wearable microwave all-dielectric metamaterial with tunable frequency selective and cloaking effects can also be designed. This reconfigurable technology will benefit many electromagnetic applications, such as frequency tuning, polarization conversion, electromagnetic absorption and shielding.
